# The T‐Type Calcium Channel CACNA1H is Required for Smooth Muscle Cytoskeletal Organization During Tracheal Tubulogenesis

**DOI:** 10.1002/advs.202308622

**Published:** 2024-10-03

**Authors:** Ziying Liu, Chunyan Lu, Li Ma, Changjiang Li, Haiyun Luo, Yiqi Liu, Xinyuan Liu, Haiqing Li, Yachao Cui, Jiahang Zeng, Natalia Bottasso‐Arias, Debora Sinner, Le Li, Jian Wang, Didier Y. R. Stainier, Wenguang Yin

**Affiliations:** ^1^ State Key Laboratory of Respiratory Disease National Clinical Research Center for Respiratory Disease Guangzhou Institute of Respiratory Health the First Affiliated Hospital of Guangzhou Medical University Guangzhou Guangdong 510182 P. R. China; ^2^ Guangzhou National Laboratory Guangzhou International Bio Island No. 9 XingDaoHuanBei Road Guangzhou Guangdong Province 510005 P. R. China; ^3^ Heart center & Department of Pediatric Surgery Guangdong Provincial Key Laboratory of Research in Structural Birth Defect Disease Guangzhou Women and Children's Medical Center Guangzhou Medical University Guangzhou Guangdong 510623 P. R. China; ^4^ Department of Thoracic Surgery Guangzhou Women and Children's Medical Center Guangzhou Medical University Guangzhou 510623 P. R. China; ^5^ Division of Neonatology and Pulmonary Biology CCHMC College of Medicine University of Cincinnati Cincinnati OH 45221 USA; ^6^ Department of Developmental Genetics Max Planck Institute for Heart and Lung Research Member of the German Center for Lung Research (DZL) 61231 Bad Nauheim Germany; ^7^ Key Laboratory of Biological Targeting Diagnosis Therapy and Rehabilitation of Guangdong Higher Education Institutes the Fifth Affiliated Hospital of Guangzhou Medical University Guangzhou 510005 P. R. China; ^8^ GMU‐GIBH Joint School of Life Sciences Guangzhou Medical University Guangzhou 511436 P. R. China

**Keywords:** Cacna1h, cytoskeleton, RhoA, smooth muscle, tracheal stenosis

## Abstract

Abnormalities of tracheal smooth muscle (SM) formation are associated with several clinical disorders including tracheal stenosis and tracheomalacia. However, the cellular and molecular mechanisms underlying tracheal SM formation remain poorly understood. Here, it is shown that the T‐type calcium channel CACNA1H is a novel regulator of tracheal SM formation and contraction. *Cacna1h* in an ethylnitrosourea forward genetic screen for regulators of respiratory disease using the mouse as a model is identified. *Cacna1h* mutants exhibit tracheal stenosis, disorganized SM and compromised tracheal contraction. CACNA1H is essential to maintain actin polymerization, which is required for tracheal SM organization and tube formation. This process appears to be partially mediated through activation of the actin regulator RhoA, as pharmacological increase of RhoA activity ameliorates the *Cacna1h*‐mutant trachea phenotypes. Analysis of human tracheal tissues indicates that a decrease in CACNA1H protein levels is associated with congenital tracheostenosis. These results provide insight into the role for the T‐type calcium channel in cytoskeletal organization and SM formation during tracheal tube formation and suggest novel targets for congenital tracheostenosis intervention.

## Graphical abstract

Defects in the T‐type calcium channel CACNA1H cause defects in airway smooth muscle formation and its cytoskeletal organization in tracheal stenosis, and pharmacological increase of RhoA activity can partially attenuate these defects in mice. Analysis of human tracheal tissues suggests that a decrease in CACNA1H proteins is associated with tracheal stenosis in patients.

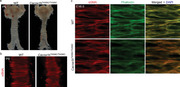

## 1 Introduction

The trachea, a tubular structure connecting the larynx, and the bronchi provides a conduit for air flow between the environment and the lung lobes. The tracheal tube is composed of internal epithelium and the surrounding mesenchyme including smooth muscle (SM), cartilage and connective tissue.^[^ ^1^ ^]^ Tracheal SM provides tissue elasticity to control its contraction, while the cartilage maintains tracheal rigidity to prevent airway collapse.^[^ ^2^ ^]^ In humans, defects in tracheal SM formation have been reported to be associated with several large airway disorders including tracheomalacia, tracheostenosis and tracheal sleeve deformity.^[^ ^3^ ^]^ Recently, two functional studies in mice have shown that altered tracheal SM organization or differentiation leads to disrupted cartilage segmentation, suggesting a critical role for the SM in tracheal tube formation.^[^ ^4^, ^5^ ^]^ However, how SM formation and function are regulated during tracheal formation remains largely unknown.

Ion channels regulate the transport of ions across the plasma membrane to control muscle contraction, synaptic transmission and neurosecretion.^[^ ^6^, ^7^, ^8^ ^]^ Several ion channels have also emerged as mediators of tissue formation including the lungs.^[^ ^4^, ^9^, ^10^, ^11^ ^]^ However, the role for calcium channels in pulmonary development is poorly understood. Calcium channels include voltage‐gated calcium channels and ligand‐gated calcium channels. Voltage‐gated calcium channels are divided into five major subtypes (L, N, P/Q, R and T) according to their electrophysiological and pharmacological characteristics.^[^ ^12^, ^13^ ^]^ T‐type voltage‐gated calcium channels include three members, CaV3.1, CaV3.2, and CaV3.3, encoded by *CACNA1G*, *CACNA1H* and *CACNA1I*, respectively.^[^ ^14^ ^]^ T‐type voltage‐gated calcium channels have been reported to be able to regulate neuronal excitability, hormone secretion, artery relaxation, platelet activation and chondrogenesis.^[^ ^15^, ^16^, ^17^ ^]^ However, whether T‐type calcium channels play a role in tracheal SM formation and function remains unknown.

Cytoskeletal organization is essential to maintain cell shape and tissue organization.^[^ ^4^, ^18^ ^]^ Recently, calcium channels incuding Piezo‐type mechanosensitive ion channels and the transient receptor potential (TRP) channel family of cation channels have been reported to modulate actin organization.^[^ ^19^, ^20^, ^21^, ^22^ ^]^ However, whether T‐type voltage‐gated calcium channels also modulate the cytoskeleton is unknown.

Here, starting with a forward genetic screen in mouse, we reveal a novel role for a specific T‐type calcium channel in tracheal SM formation and function to drive tracheal tube formation, at least in part via the control of RhoA‐mediated cytoskeletal organization. Analysis of human tracheal tissues uncover that reduced levels of CACNA1H is associated with symptoms of tracheostenosis in humans.

## 2 Results

### 2.1 *Cacna1h^T4306C/T4306C^* Mice Exhibit Tracheal Formation Defects

**Figure 1:**
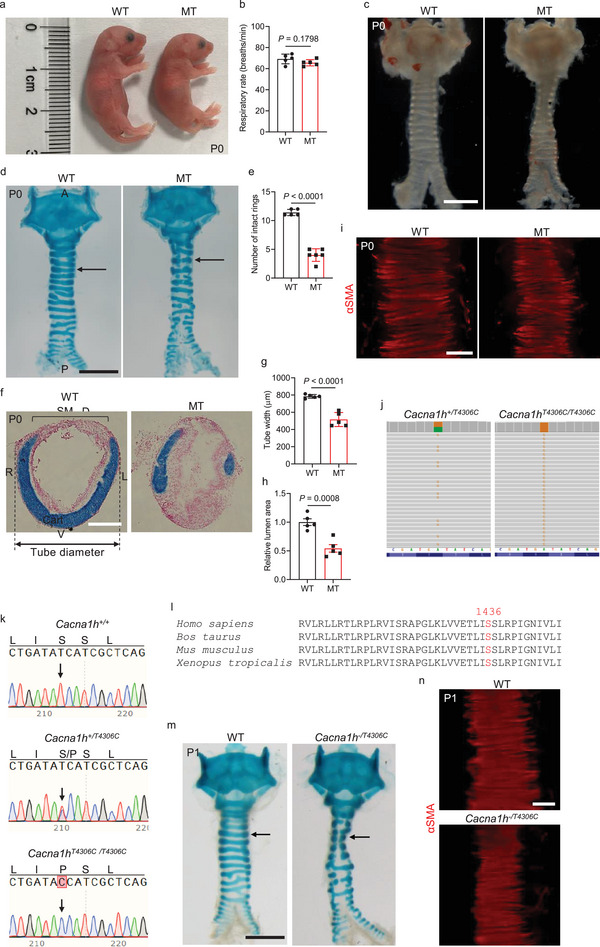
*Cacna1h^T4306C/T4306C^* mice exhibit tracheal tube formation defects. a) Representative gross morphology of P0 WT (n = 5) and mutants (n = 5). b) Quantification of P0 WT (n = 5) and mutant (n = 5) respiratory rate. c) Representative images of ventral views of P0 WT (n = 5) and mutant (n = 6) tracheae. d) Representative image of ventral views of wholemount tracheae stained with alcian blue from P0 WT (n = 5) and mutants (n = 6). Arrows point to tracheal cartilage rings. e) Quantification of the number of intact tracheal cartilage rings from P0 WT (n = 5) and mutants (n = 6). f) Representative images of transverse sections of tracheae stained with alcian blue and nuclear fast red from P0 WT (n = 5) and mutants (n = 5). g) Quantification of P0 WT (n = 5) and mutant (n = 5) tracheal tube diameter. h) Quantification of P0 WT (n = 5) and mutant (n = 5) tracheal lumen area. i) Representative images of dorsal views of wholemount tracheae stained for αSMA (red) from P0 WT (n = 5) and mutants (n = 6). j) Whole‐exome sequencing of control (*Cacna1h* heterozygous, n = 2) and mutant (*Cacna1h* homozygous, n = 2) genomic DNA. Green indicates the WT nucleotide A. Orange indicates the mutant nucleotide G. k) Sequence of WT and mutant genomic DNA around the lesion. DNA sequence chromatograms show TCA for serine in WT (n = 98) (upper panel), TCA and CCA in heterozygous mutants (n = 201) (middle panel), and CCA for proline in homozygous mutants (n = 102) (lower panel). Arrows point to the mutation site. l) Evolutionary conservation of the p.S1436 residue in vertebrates. m) Representative images of ventral views of wholemount tracheae stained with alcian blue from P1 WT (n = 7) and *Cacna1h^+/T4306C^* double heterozygous animals (n = 7). Arrows point to tracheal cartilage rings. n) Representative images of dorsal views of wholemount tracheae stained for αSMA (red) from P1 WT (n = 5) and *Cacna1h^+/T4306C^* double heterozygous animals (n = 5). Scale bars: 1000 µm c,d,m), 200 µm f,i,n). Unpaired Student's *t*‐test. Data are mean ± s.d. WT, Wild type; MT, Mutant; A, Anterior; P, Posterior; D, Dorsal; V, Ventral; L, Left; R, Right; Cart, Cartilage; SM, Smooth muscle.

**Figure 2:**
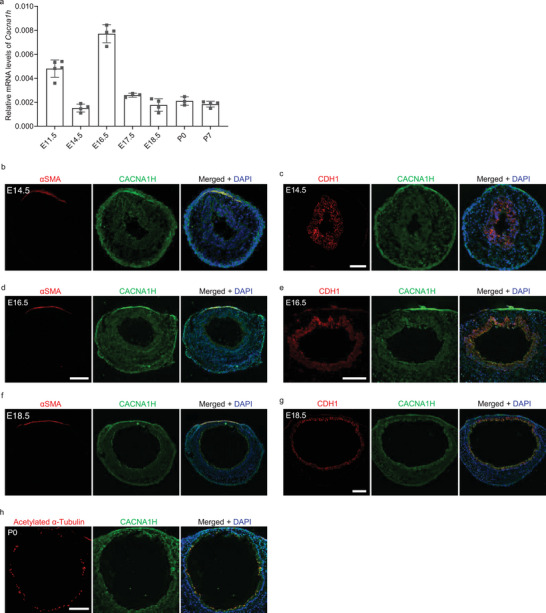
*Cacna1h* is expressed in SM and epithelial cells in the trachea. a) RT‐qPCR analysis of *Cacna1h* mRNA levels in the trachea at several embryonic and postnatal stages. Immunostaining for αSMA (red) and CACNA1H (green) and DAPI staining (blue) of transverse sections of E14.5 (n = 7) b), E16.5 (n = 7) d) and E18.5 (n = 7) f) WT tracheae. Immunostaining for CDH1 (red) and CACNA1H (green) and DAPI staining (blue) of transverse sections of E14.5 (n = 7) c), E16.5 (n = 7) e) and E18.5 (n = 7) g) WT tracheae. h) Immunostaining for acetylated α‐Tubulin (red) and CACNA1H (green) and DAPI staining (blue) of transverse sections of P0 tracheae (n = 3). Scale bars: 100 µm d–h), 50 µm b,c).

To identify novel regulators of respiratory disease, we conducted a large‐scale forward genetic screen using ethylnitrosourea (ENU) mutagenesis.^[^ ^4^ ^]^ One of the recessive mutants identified in this screen exhibits WT (wild type)‐like respiratory rate (**Figure** **1**a,b) with tracheal stenosis characterized by a narrowed trachea with fractured cartilage rings (Figure 1c–h) but WT‐like tracheal tube length (Figure S1a, Supporting Information). These mutant animals are born in the expected Mendelian ratio, suggesting that this mutation does not cause embryonic lethality. Interestingly, disorganized SM, characterized by the narrowing of SM stripes, was observed in the mutants (Figure 1i). To identify the phenotype‐causing mutation, we performed whole‐exome sequencing of G4 genomic DNA samples, and identified *Cacna1h*, which encodes a T‐type voltage‐gated calcium channel, as a candidate gene (Figure 1j). Next, we carried out genetic linkage analysis by genotyping 178 G4, G5, G6 and G7 mutant animals, and found complete linkage between the tracheal phenotype and the *Cacna1h^T4306C/T4306C^* allele (Figure 1k). This identified allele carries a mutation that causes a serine‐to‐proline substitution at the highly conserved 1436th residue (c.4306T>C (p.Ser1436Pro)) (Figure 1l). We then performed a complementation test by crossing mice carrying the ENU‐induced *Cacna1h* allele (*Cacna1h^T4306C/+^*) with mice carrying a *Cacna1h* deletion allele,^[^ ^23^ ^]^ and found that complementation did not occur in the *Cacna1h^−/T4306C^* double heterozygous animals (Figure 1m,n), indicating that loss of *Cacna1h* function is likely responsible for the observed tracheal phenotypes. To further test the role for *Cacna1h* in tracheal development, we analyzed tracheal formation in *Cacna1h^−/−^* mice. *Cacna1h^−/−^* mice exhibited a narrowed trachea with fractured cartilage rings (Figure S2a–c, Supporting Information), similar to the phenotypes reported before.^[^ ^16^ ^]^ We also observed narrowed SM stripes in *Cacna1h^−/−^* tracheae which has not been reported before (Figure S2d, Supporting Information). We then examined the spatial and temporal expression pattern of *Cacna1h* in the developing mouse trachea. *Cacna1h* mRNA was dynamically expressed in E11.5‐P7 tracheae (**Figure** **2**a). We also examined CACNA1H protein expression at E14.5. CACNA1H was clearly detected in the trachea including the SM and epithelium (Figure 2b,c). At E16.5 and E18.5, CACNA1H was still expressed in tracheal SM cells and epithelial cells (Figure 2d–g). We also observed that CACNA1H was expressed in acetylated α‐tubulin^+^ multiciliate cells at P0 (Figure 2h). Altogether, these results suggest that *Cacna1h* regulates tracheal SM development and tracheal tube formation.

### 2.2 *Cacna1h^T4306C/T4306C^* Mice Display Defects in SM Formation and Cartilage Condensation

**Figure 3:**
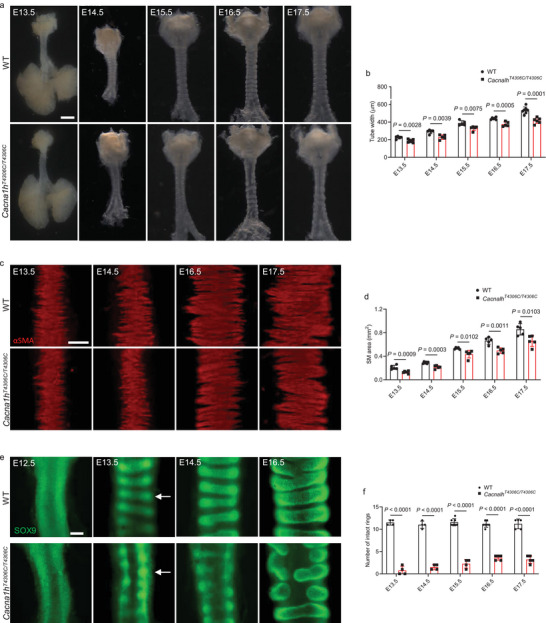
*Cacna1h^T4306C/T4306C^* mice exhibit defects in tracheal expansion, SM organization, and mesenchymal condensation. a) Representative images of ventral views of WT (n = 5‐7) and *Cacna1h^T4306C/T4306C^* (n = 5‐7) tracheae at several embryonic stages. b) Quantification of WT (n = 5‐7) and *Cacna1h^T4306C/T4306C^* (n = 5‐7) tracheal tube width. c) Immunostaining for αSMA (red) in dorsal views of WT (n = 5‐6) and *Cacna1h^T4306C/T4306C^* (n = 5‐6) tracheae at several embryonic stages. d) Quantification of WT (n = 5‐6) and *Cacna1h^T4306C/T4306C^* (n = 5‐6) SM area. e) Immunostaining for SOX9 (green) in ventral views of WT (n = 4‐7) and *Cacna1h^T4306C/T4306C^* (n = 4‐5) tracheae at several embryonic stages. Arrows point to tracheal mesenchymal condensations. f) Quantification of intact tracheal mesenchymal condensations in WT (n = 4‐7) and *Cacna1h^T4306C/T4306C^* (n = 4‐5) animals. Scale bars: 500 µm a), 100 µm c,e). Unpaired Student's *t*‐test. Data are mean ± s.d.

To examine the defects in *Cacna1h^T4306C/T4306C^* trachea formation in detail, we performed a systematic analysis of tracheal tube development. Starting at E13.5, we observed that *Cacna1h^T4306C/T4306C^* tracheae were slightly narrowed compared with WT (**Figure** **3**a,b), indicating that impaired tracheal tube expansion occurs after tracheal cartilage ring formation and SM differentiation, which start at E13.5 and E11.5, respectively.^[^ ^2^, ^4^ ^]^ The narrowed trachea phenotype appeared more obvious starting at E14.5 (Figure 3a,b). Since defects in SM formation can affect airway width,^[^ ^5^, ^24^ ^]^ we analyzed tracheal SM development. Disorganized SM stripes of decreased area were observed in E13.5 *Cacna1h^T4306C/T4306C^* tracheae and became more noticeable starting at E14.5 (Figure 3c,d). Since altered cartilage formation can also lead to narrowed tracheae,^[^ ^25^, ^26^ ^]^ we examined tracheal cartilage development. At E12.5, SOX9^+^ mesenchymal cells have not yet condensed in WT or *Cacna1h^T4306C/T4306C^* tracheae (Figure 3e,f). At E13.5, a clear pattern of condensed SOX9^+^ mesenchymal cells resembling cartilaginous rings can be readily distinguished in WT, whereas such condensations were barely detected in *Cacna1h^T4306C/T4306C^* tracheae (Figure 3e,f). Since *Cacna1h^T4306C/T4306C^* mice survived into adulthood, we next examined cartilage rings and SM in adult tracheas. We found that 6‐month‐old *Cacna1h^T4306C/T4306C^* animals also exhibited narrowed tracheae (Figure S3a,b, Supporting Information), fracture of cartilage rings (Figure S3c, Supporting Information) and disorganized SM (Figure S3d,e, Supporting Information). The effect on SM organization and mesenchymal condensation appeared to be specific, as we did not find significant differences between WT and mutant animals in terms of SM cell proliferation (Figure S4a,b, Supporting Information), SOX9^+^ mesenchymal cell proliferation (Figure S4c,d, Supporting Information), or tracheal cell apoptosis (Figure S4e,f, Supporting Information). In addition, we observed no obvious changes in differentiation of KRT5^+^ basal cells (Figure S5a, Supporting Information), SCGB1A1^+^ club cells (Figure S5b, Supporting Information) or acetylated alpha‐tubulin^+^ multiciliated cells (Figure S5c, Supporting Information) in *Cacna1h^T4306C/T4306C^* tracheae. Since *Shh*, *Fgf10* and *Fgfr2b* modulates tracheal mesenchymal formation,^[^ ^27^, ^28^, ^29^, ^30^ ^]^ we examined for changes in expression of these genes. *Cacna1h^T4306C/T4306C^* tracheae exhibited no significant differences in expression levels of *Shh*, *Fgf10* or *Fgfr2b* (Figure S6a–c, Supporting Information), indicating that *Cacna1h* signals via a Shh, Fgf10 and *Fgfr2b* independent pathway to direct tracheal formation. Altogether, these data suggest that CACNA1H promotes SM formation and mesenchymal condensation necessary for tracheal tube formation.

### 2.3 *Cacna1h* Mediates SM CELL Alignment and Cell Shape

**Figure 4:**
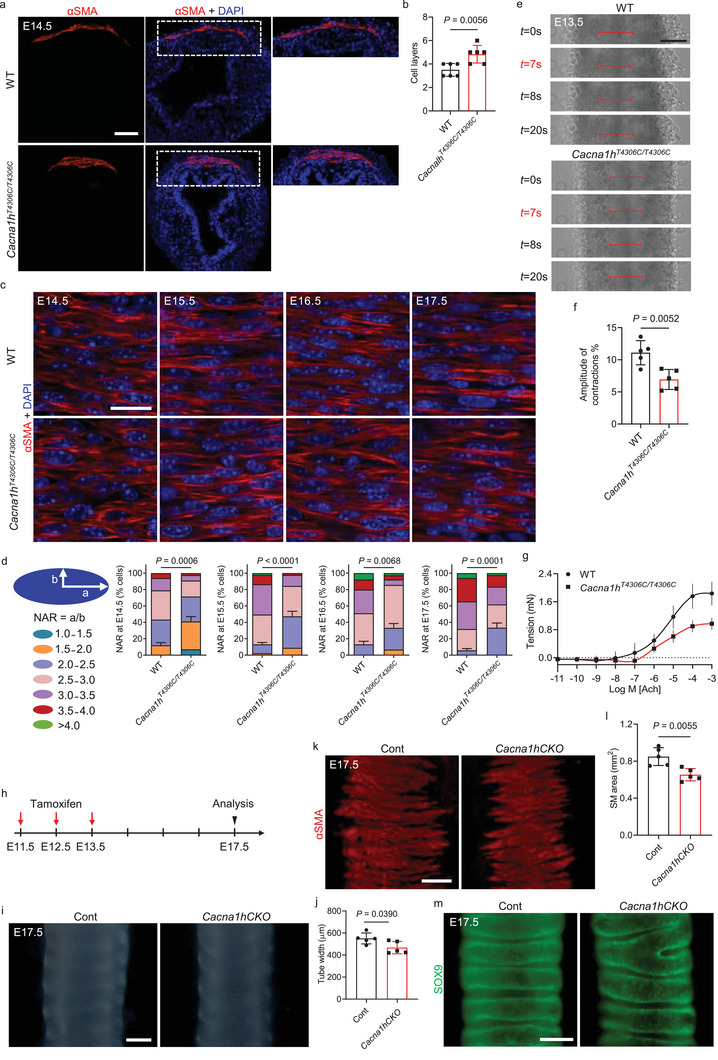
*Cacna1h* orchestrates SM cell alignment and shape. a) Immunostaining for αSMA (red) and DAPI staining (blue) of transverse sections of E14.5 WT (n = 6) and *Cacna1h^T4306C/T4306C^* (n = 6) tracheae. b) Quantification of E14.5 WT (n = 6) and *Cacna1h^T4306C/T4306C^* (n = 6) tracheal SM cell layers. c) Immunostaining for αSMA (red) and DAPI staining (blue) in dorsal views of WT (n = 4) and *Cacna1h^T4306C/T4306C^* (n = 4) tracheae at several embryonic stages. d) Quantification of WT (n = 4) and *Cacna1h^T4306C/T4306C^* (n = 4) tracheal SM cell nuclear aspect ratio (NAR) at several embryonic stages. e) Dorsal views of time‐lapse images of spontaneous contractions of E13.5 WT (n = 5) and *Cacna1h^T4306C/T4306C^* (n = 5) tracheae. Lines indicate tracheal smooth muscle width. f) Quantification of the amplitude of contractions of E13.5 WT (n = 5) and *Cacna1h^T4306C/T4306C^* (n = 5) tracheae. g) Tracheal tension of P14 WT (n = 4) and *Cacna1h^T4306C/T4306C^* (n = 4) tracheae. h) Timeline for tamoxifen administration. i) Representative images of ventral views of E17.5 control (n = 5) and *Cacna1hCKO* (n = 5) tracheae. j) Quantification of E17.5 control (n = 5) and *Cacna1hCKO* (n = 5) (n = 5) tracheal tube width. k) Immunostaining for αSMA (red) in dorsal views of E17.5 control (n = 5) and *Cacna1hCKO* (n = 5) tracheae. l) Quantification of control (n = 5) and *Cacna1hCKO* (n = 5) SM area. m) Immunostaining for SOX9 (green) in ventral views of E17.5 control (*n*  =  5) and *Cacna1hCKO* (*n*  =  5) tracheas. Scale bars: 200 µm i,m), 100 µm j), 50 µm a,e), 20 µm c). Unpaired Student's *t*‐test. Data are mean ± s.d. Ach, acetylcholine; Cont, *Myh11‐CreER^T2^;Cacna1h* ^+^ *^/^* ^+^; *Cacna1hCKO*, *Myh11‐CreER^T2^;Cacna1h^flox/flox^*.

To further investigate the effects of *Cacna1h^T4306C/T4306C^* on tracheal SM organization, we analyzed SM cell alignment and shape in WT and *Cacna1h^T4306C/T4306C^* tracheae. Tracheal SM cells differentiate at E11.5 and can acquire well‐developed spindle shapes to circumferentially align the tube after E14.5.^[^ ^2^, ^26^ ^]^ *Cacna1h^T4306C/T4306C^* SM cells aligned in a direction perpendicular to that of tracheal elongation at E14.5, similar to WT animals (**Figure** **4**a). Interestingly, *Cacna1h^T4306C/T4306C^* SM cells aligned into 4–6 layers compared with 3–4 layers in WT at E14.5 (Figure 4a,b), suggesting that lack of *Cacna1h* function disrupts the cell layer alignment of SM, which leads to thickened and narrowed SM stripes. Inactivation of ion channels including potassium channels and chloride channels can lead to changes in tracheal SM cell shape.^[^ ^4^, ^10^ ^]^ Next, we tested whether *Cacna1h* was required for SM cell shape acquisition*. Cacna1h^T4306C/T4306C^* tracheae exhibited altered SM cell shape characterized by reduced nuclear aspect ratio in E14.5‐17.5 tracheae (Figure 4c,d). Ion channels are essential for tracheal contractions.^[^ ^4^ ^]^ Interestingly, *Cacna1h^T4306C/T4306C^* tracheae exhibited reduced amplitude of spontaneous contractions compared to WT at E13.5 (Figure 4e,f; Movie S1, Supporting Information). In addition, *Cacna1h^T4306C/T4306C^* tracheae exhibited significantly reduced contractile responses elicited by acetylcholine compared with WT (Figure 4g). Collectively, these data indicate that CACNA1H is required to coordinate SM cell alignment and shape as well as contractility to direct tracheal tissue architecture.

To further test whether SM specific deletion of *Cacna1h* could lead to defects in SM formation, we generated a *Cacna1h^flox^* allele (Figure S7, Supporting Information) and used the SM‐specific Cre mouse line *Myh11*‐*CreER^T2^*, which induces efficient recombination in airway SM cells.^[^ ^4^, ^31^ ^]^ We conditionally deleted *Cacna1h* in SM by intraperitoneal tamoxifen injections for 3 consecutive days (2 mg per day) from E11.5 to E14.5 (Figure 4h). Mice with SM‐specific *Cacna1h* deletion (*Myh11*‐*CreER^T2^;Cacna1h^flox/flox^*: *Cacna1hCKO*) exhibited a narrowed trachea with reduced SM stripe area (Figure 4i–l), similar to those observed in *Cacna1h^T4306C/T4306C^* animals (Figure 3c,d). Interestingly, *Cacna1hCKO* tracheae showed WT‐like cartilage patterning (Figure 4m). Altogether, these data indicate that *Cacna1h* functions in a cell‐autonomous fashion to drive tracheal SM formation to promote tracheal expansion.

Both the trachea and esophagus are derived from a common foregut tube and elongate in parallel during tracheal tubulogenesis. Next, we sought to determine whether *Cacna1h* was also required for esophageal tube formation. We measured P0 esophagi and found no significant difference in tube width between *Cacna1h^T4306C/T4306C^* and WT mice (Figure S8a,b, Supporting Information). We also examined esophageal SM morphology. SM cells locate circumferentially in the outer layers of the esophageal wall.^[^ ^4^, ^26^ ^]^ Unlike in the trachea, we observed no obvious differences in esophageal SM organization (Figure S8c, Supporting Information) between *Cacna1h^T4306C/T4306C^* and WT mice. We also analyzed the expression pattern of CACNA1H in the developing esophagus and found that CACNA1H was highly expressed in E14.5 esophageal SM cells and epithelial cells (Figure S8d,e, Supporting Information). These data indicate that CACNA1H is dispensable for esophageal tube development or SM formation.

### 2.4 *Cacna1h* Mediates Actin Organization in SM Cells

**Figure 5:**
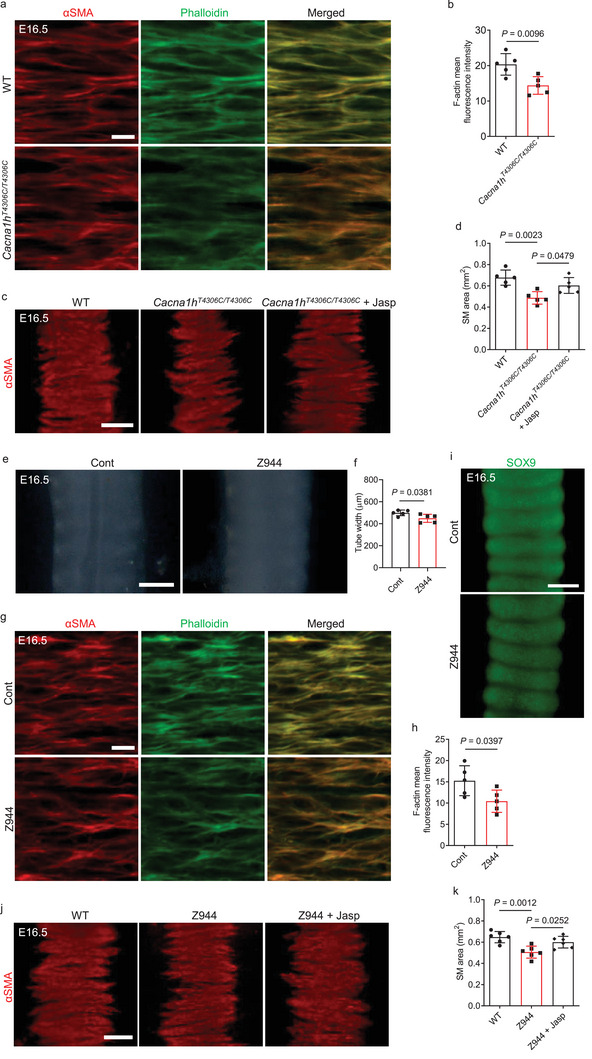
*Cacna1h* regulates actin organization in SM cells. a) Immunostaining for αSMA (red) and phalloidin (green), and DAPI (blue) staining in dorsal views of E16.5 WT (n = 5) and *Cacna1h^T4306C/T4306C^* (n = 5) tracheae. b) Quantification of mean phalloidin fluorescence intensity in E16.5 WT (n = 5) and *Cacna1h^T4306C/T4306C^* (n = 5) tracheal SM cells. c) Immunostaining for αSMA (red) in dorsal views of E16.5 WT (n = 5) and *Cacna1h^T4306C/T4306C^* (n = 5) tracheae after a 48 h DMSO treatment, and *Cacna1h^T4306C/T4306C^* tracheae (n = 5) after a 48 h 300 nM jasplakinolide treatment. d) Quantification of SM area of E16.5 WT (n = 5) and *Cacna1h^T4306C/T4306C^* (n = 5) tracheae after a 48 h DMSO treatment, and *Cacna1h^T4306C/T4306C^* tracheae (n = 5) after a 48 h 300 nM jasplakinolide treatment. e) Representative images of ventral views of E16.5 tracheae after a 48 h DMSO (n = 5) or 2 µM Z944 (n = 5) treatment. f) Quantification of E16.5 tracheal tube width after a 48 h DMSO (n = 5) or 2 µM Z944 (n = 5) treatment. g) Immunostaining for αSMA (red) and phalloidin staining (green) in dorsal views of E16.5 tracheae after a 48 h DMSO (n = 5) or 2 µM Z944 (n = 5) treatment. h) Quantification of mean phalloidin fluorescence intensity in E16.5 tracheal SM cells after a 48 h DMSO (n = 5) or 2 µM Z944 (n = 5) treatment. i) Immunostaining for SOX9 (green) in ventral views of E16.5 tracheae after a 48 h DMSO (n = 5) or 2 µM Z944 (n = 5) treatment. j) Immunostaining for αSMA (red) in dorsal views of E16.5 WT tracheae after a 48 h DMSO (n = 5) or 2 µM Z944 (n = 5) treatment, or a 48 h 2 µM Z944 plus 36 h 300 nM jasplakinolide (n = 5) treatment. k) Quantification of SM area of E16.5 WT tracheae after a 48 h DMSO (n = 6) or 2 µM Z944 (n = 6) treatment, or a 48 h 2 µM Z944 plus 36 h 300 nM jasplakinolide (n = 6) treatment. Scale bars: 200 µm e,i), 100 µm c,j), 10 µm a,g). Unpaired Student's *t*‐test b,f,h); one‐way ANOVA with Dunnett's multiple‐comparison correction d,k). Data are mean ± s.d. Cont, Control; Jasp, jasplakinolide.

**Figure 6:**
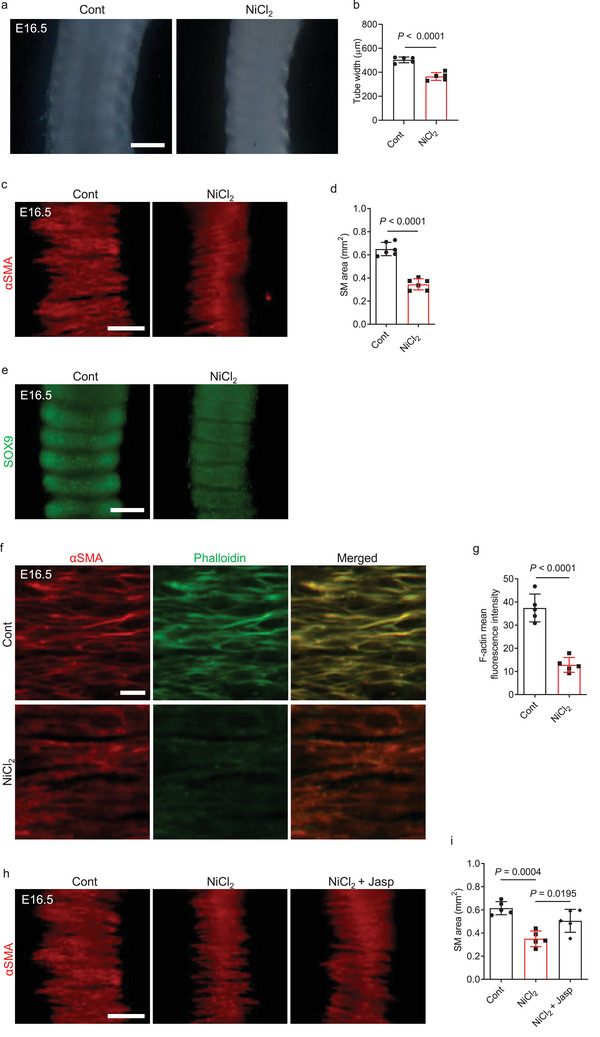
NiCl_2_ treatment leads to narrowed tracheal SM stripes and reduced F‐actin levels. a) Representative images of ventral views of E16.5 tracheae after a 48 h DMSO (n = 5) or 30 µM NiCl_2_ (n = 5) treatment. b) Quantification of E16.5 tracheal tube width after a 48 h DMSO (n = 5) or 30 µM NiCl_2_ (n = 5) treatment. c) Immunostaining for αSMA (red) in dorsal views of E16.5 WT tracheae after a 48 h ddH_2_O (n = 6) or 30 µM NiCl_2_ (n = 6) treatment. d) Quantification of SM area of E16.5 WT after a 48 h ddH_2_O (n = 6) or 30 µM NiCl_2_ (n = 6) treatment. e) Immunostaining for SOX9 (green) in ventral views of E16.5 tracheae after a 48 h DMSO (n = 5) or 30 µM NiCl_2_ (n = 5) treatment. f) Immunostaining for αSMA (red) and phalloidin staining (green) in dorsal views of E16.5 tracheae after a 48 h ddH_2_O (n = 5) or 30 µM NiCl_2_ (n = 5) treatment. g) Quantification of mean phalloidin fluorescence intensity in E16.5 tracheal SM cells after a 48 h ddH_2_O (n = 5) or 30 µM NiCl_2_ (n = 5) treatment. h) Immunostaining for αSMA (red) in dorsal views of E16.5 WT tracheae after a 48 h ddH_2_O (n = 5) or 30 µM NiCl_2_ (n = 5) treatment, or a 48 h 30 µM NiCl_2_ plus 36 h 300 nM jasplakinolide (n = 5) treatment. i) Quantification of SM area of E16.5 WT tracheae after a 48 h ddH_2_O (n = 5) or 30 µM NiCl_2_ (n = 5) treatment, or a 48 h 30 µM NiCl_2_ plus 36 h 300 nM jasplakinolide (n = 5) treatment. Scale bars: 200 µm a,e), 100 µm c,h), 10 µm f). Unpaired Student's *t*‐test b,d,g); one‐way ANOVA with Dunnett's multiple‐comparison correction i). Data are mean ± s.d.

Actin filaments (F‐actin) provide mechanical support and affect cell shape and tissue structures.^[^ ^4^, ^32^ ^]^ Several calcium channels including Piezo‐type mechanosensitive ion channels and the TRP channels have been reported to modulate the actin cytoskeleton.^[^ ^19^, ^20^, ^21^, ^22^ ^]^ We hypothesized that *Cacna1h^T4306C/T4306C^* might lead to actin depolymerization in tracheal SM cells, and consequently to alteration of SM formation. We examined F‐actin content by phalloidin staining, and found that *Cacna1h^T4306C/T4306C^* tracheae exhibited reduced F‐actin levels in SM cells compared with WT (**Figure** **5**a,b). Interestingly, *Cacna1h^T4306C/T4306C^* tracheae, after treatment with 300 nM jasplakinolide, a small molecule that promotes actin polymerization and stabilizes actin filaments,^[^ ^33^, ^34^ ^]^ exhibited partially rescued SM phenotypes compared with controls (Figure 5c,d). Interestingly, a treatment with 2 µM Z944, a selective T‐type calcium channel blocker^[^ ^35^ ^]^ also caused a narrowed trachea with decreased F‐actin levels in tracheal SM cells (Figure 5e–h) and in primary human airway SM cells (Figure S9a,b, Supporting Information) but WT‐like cartilage patterning (Figure 5i). Z944‐treated WT tracheae, after treatment with 300 nM jasplakinolide, exhibited partially rescued SM phenotypes compared with controls (Figure 5j,k). These results indicate that reduced actin polymerization in *Cacna1h^T4306C/T4306C^* tracheae partially accounts for the SM formation defects. Ni^2+^ is found to selectively inhibit CACNA1H T‐type calcium channels at low concentrations (<50 µM)^[^ ^36^, ^37^ ^]^ An *ex vivo* treatment with 30 µM NiCl_2_ led to a narrowed trachea with a narrowing of SM stripes (**Figure** **6**a–d) but WT‐like cartilage patterning (Figure 6e). Notably, treatment with 30 µM NiCl_2_ also caused decreased F‐actin levels in tracheal SM cells and in primary human airway SM cells (Figure 6f,g; Figure S9c,d, Supporting Information). These defects appeared more severe than those observed in *Cacna1h^T4306C/T4306C^* animals, suggesting that Ni^2+^ may affect addtional target(s) involved in tracheal SM formation. Interestingly, NiCl_2_‐treated WT tracheae, after treatment with 300 nM jasplakinolide, also exhibited partially rescued SM phenotypes compared with controls (Figure 6h,i). Altogether, these results indicate that reduced actin polymerization in *Cacna1h^T4306C/T4306C^* tracheae partially accounts for the defects in SM formation.

### 2.5 RhoA as a Mediator of *Cacna1h* Function in SM Cells

**Figure 7:**
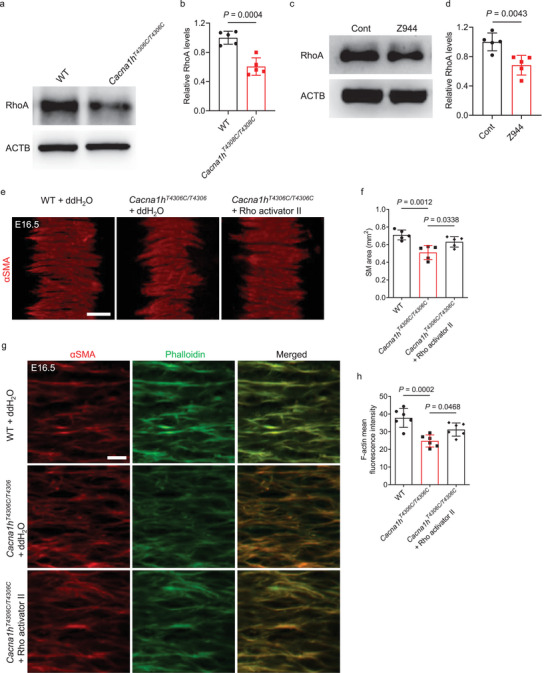
RhoA as a mediator of *Cacna1h* function in tracheal SM cells. a) Western blotting for RhoA and ACTB in P0 WT (n = 5) and *Cacna1h^T4306C/T4306C^* (n = 5) tracheae. b) Quantification of relative RhoA levels in P0 WT (n = 5) and *Cacna1h^T4306C/T4306C^* (n = 5) tracheae. c) Western blotting for RhoA and ACTB in P0 tracheae after a 48 h DMSO (n = 5) or 2 µM Z944 (n = 5) treatment. d) Quantification of relative RhoA levels in P0 tracheae after a 48 h DMSO (n = 5) or 2 µM Z944 (n = 5) treatment. e) Immunostaining for αSMA (red) in dorsal views of E16.5 WT (n = 5) and *Cacna1h^T4306C/T4306C^* (n = 5) tracheae after a 48 h ddH_2_O treatment, and *Cacna1h^T4306C/T4306C^* (n = 5) tracheae after a 2 h 0.005 µg ml^−1^ Rho Activator II treatment plus 46 h ddH_2_O treatment. f) Quantification of SM area of E16.5 WT (n = 5) and *Cacna1h^T4306C/T4306C^* (n = 5) tracheae after a 48 h ddH_2_O treatment, and *Cacna1h^T4306C/T4306C^* (n = 5) tracheae after a 2 h 0.005 µg ml^−1^ Rho Activator II treatment plus 46 h ddH_2_O treatment. g) Immunostaining for αSMA (red) and phalloidin staining (green) in dorsal views of E16.5 WT (n = 5) and *Cacna1h^T4306C/T4306C^* (n = 5) tracheae after a 48 h ddH_2_O treatment, and *Cacna1h^T4306C/T4306C^* (n = 5) tracheae after a 2 h 0.005 µg ml^−1^ Rho Activator II treatment plus 46 h ddH_2_O treatment. h) Quantification of mean phalloidin fluorescence intensity in SM cells in E16.5 WT (n = 6) and *Cacna1h^T4306C/T4306C^* (n = 6) tracheae after a 48 h ddH_2_O treatment, and *Cacna1h^T4306C/T4306C^* (n = 6) tracheae after a 2 h 0.005 µg ml^−1^ Rho Activator II treatment plus 46 h ddH_2_O treatment. Scale bars: 100 µm e), 10 µm g). Unpaired Student's *t*‐test b,d); one‐way ANOVA with Dunnett's multiple‐comparison correction f,h). Data are mean ± s.d.

We sought to further understand how CACNA1H influences actin organization in tracheal SM cells. Expression of RhoA, a member of the Rho family of small Ras‐like GTPases that can promote actin organization,^[^ ^20^ ^]^ has been reported to be regulated by calcium concentration and calcium channels.^[^ ^38^ ^]^ We thus examined RhoA protein levels and found that they were significantly reduced in *Cacna1h^T4306C/T4306C^* tracheae compared with WT (**Figure** **7**a,b). After 2 µM Z944 treatment, WT tracheae also exhibited significantly reduced RhoA protein levels compared with controls (Figure 7c,d). In addition, siRNA‐mediated *Cacna1h* knockdown also caused reduced F‐actin levels (Figure S9e,f, Supporting Information) and RHOA (Figure S9g–i, Supporting Information) in primary human airway SM cells compared with controls. Interestingly, *Cacna1h^T4306C/T4306C^* tracheae, after treatment with Rho Activator II, a modulator that can increase the activity RhoA^[^ ^39^ ^]^ and promote actin assembly,^[^ ^40^ ^]^ exhibited partially rescued SM cell phenotypes (Figure 7e,f) and F‐actin levels (Figure 7g,h) compared with controls. To examine global changes in gene expression in *Cacna1h^T4306C/T4306C^* tracheae during embryonic development, we performed bulk RNA‐Seq in E16.5 and E17.5 tracheae. We found that 131 genes were downregulated and 131 were upregulated in E16.5 tracheae (Figure S10a,c and Table S1, Supporting Information), and 198 genes were downregulated and 227 were upregulated in E17.5 tracheae (Figure S10b,c and Table S1, Supporting Information). Interestingly, 53 common genes were differentially regulated in *Cacna1h^T4306C/T4306C^* tracheae compared with WT at E16.5 and E17.5 (Figure S10c and Table S1, Supporting Information). These genes were involved in several biological processes, cellular components and molecular functions including muscle contraction and cytoskeleton (Figure S10d,e and Table S2, Supporting Information). Collectively, these results indicate that reduced RhoA levels in *Cacna1h^T4306C/T4306C^* tracheae partially account for the SM organization phenotypes via its action on actin polymerization.

### 2.6 CACNA1H Levels are Decreased in the Tracheae of Human Tracheostenosis Patients

**Figure 8:**
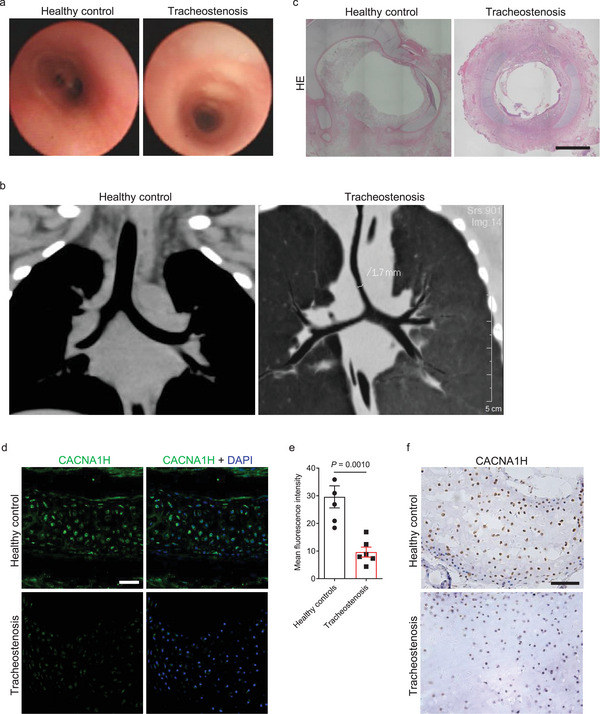
CACNA1H levels are decreased in the tracheae of human tracheostenosis patients. a) Representative bronchoscopic views of tracheae from healthy controls (n = 6) and tracheostenosis patients (n = 6). b) Representative computed tomography morphology of tracheae from healthy controls (n = 6) and tracheostenosis patients (n = 6). c) Representative images of transverse sections of tracheae stained with hematoxylin and eosin from healthy controls (n = 5) and tracheostenosis patients (n = 5). d) Immunostaining for CACNA1H (green) and DAPI staining (blue) of transverse sections of tracheae from healthy controls (n = 5) and tracheostenosis patients (n = 6). e) Quantification of mean fluorescence intensity of CACNA1H immunostaining in the tracheae from healthy controls (n = 5) and tracheostenosis patients (n = 6). f) Immunohistochemical staining for CACNA1H of transverse sections of tracheae from healthy controls (n = 5) and tracheostenosis patients (n = 6). Scale bars: 500 µm c), 100 µm f), 50 µm d). Unpaired Student's *t*‐test. Data are mean ± s.d.

Since *Cacna1h^T4306C/T4306C^* mice exhibited a narrowed trachea, phenotypes similar to those observed in tracheostenosis patients (**Figure** **8**a–c), we examined CACNA1H expression in tracheae from healthy controls and tracheostenosis patients (Table S3, Supporting Information). We observed that CACNA1H levels were significantly decreased in the cartilage regions of tracheae from tracheostenosis patients compared with healthy controls (Figure 8d–f). These results suggest that a decrease in CACNA1H is associated with, and may contribute to, tracheostenosis in humans.

## 3 Discussion

Tracheal tubulogenesis defects have been reported to lead to pulmonary disease, such as primary tracheomalacia and congenital tracheostenosis.^[^ ^3^, ^41^ ^]^ One type of tracheostenosis is complete tracheal ring deformity (CTRD), a congenital condition characterized by circumferentially continuous or nearly continuous cartilaginous tracheal rings and variable degrees of tracheal stenosis and/or shortening together with defects in trachealis SM formation.^[^ ^3^ ^]^ Given the importance of SM in tracheal development and disorders, it is important to identify specific regulators and reveal their roles during SM formation. *Cacna1h^T4306C/T4306C^* mice, identified in our ENU‐mutagenesis screen, exhibit severe disorganized tracheal SM, characterized by the narrowing of SM stripes, and tracheal stenosis. Notably, the *Myh11‐CreER^T2^;Cacna1h^flox/flox^* analysis also uncovers this function, where *Cacna1h* cell‐autonomously controls SM structure and tracheal formation. In addition, SM organization does not appear to be affected in *Foxg1^Cre^;Sox9^flox/flox^* tracheae, where cartilage formation is specifically disrupted.^[^ ^2^ ^]^ It thus appears that tracheal SM formation is independent of cartilage development in several contexts.

Absence of airway SM cell differentiation leads to reduced airway diameter in the lungs in *Tbx4rtTA;tetOcre;Myocd^flox/flox^* mice.^[^ ^5^ ^]^ Another study reveals that *Gli2^−/−^* and *Gli2^−/−^ Gli3^−/−^* mice display hypoplastic tracheae, which are characterized by narrowed tracheae with malformed cartilaginous rings.^[^ ^42^ ^]^ Mesenchenal but not epithelial deletion of *Smoothened* and activation of *Gli3* cause tracheal cartilage formation defects and reduced trachealis SM mass together with tracheal stenosis or tracheomalacia.^[^ ^43^, ^44^ ^]^ It would be interesting to examine whether *Gli2^−/−^* and *Gli2^−/−^ Gli3^−/−^* mutants exhibit tracheal SM defects, or SM specific deletion of *Gli2* and *Gli3* exhibits tracheal stenosis and SM disorganization. *Notum* and *Kcnj13* mutant mice also display narrowed tracheae together with diminished and disorganized SM, respectively.^[^ ^4^, ^45^ ^]^ It thus appears that defects in tracheal SM formation is widely associated with, and may contribute to tracheal stenosis or tracheomalacia in several contexts.

Inactivation of the calcium‐activated chloride channel TMEM16A or the potassium channel KCNJ13 leads to altered tracheal SM cell shape and organization.^[^ ^4^, ^10^ ^]^ Deletion of CFTR, a chloride ion channel causes reduced tracheal SM cell mass.^[^ ^46^ ^]^ These phenotypes are all found in *Cacna1h^T4306C/T4306C^* tracheae. It thus appears that multiple ion channels regulate tracheal SM structure. It will be interesting to test whether these ion channels genetically interact to modulate SM formation.

Low concentrations of NiCl_2_ (<50 µM) are reported to selectively block CACNA1H over the other T‐type calcium channels.^[^ ^36^, ^37^ ^]^ A 30 µM NiCl_2_ treatment to specifically inhibit CACNA1H can reduce platelet activation as assessed by measuring platelet secretion and integrin α_IIb_β3 activation.^[^ ^17^ ^]^ Severity of NiCl_2_ treatment‐caused phenotypes of reduced platelet activation appears similar to those observed in *Cacna1h^−/−^* platelets.^[^ ^17^ ^]^ However, our data show that a treatment of 30 µM NiCl_2_ causes more severe narrowed tracheal SM and defects in actin organization than those found in *Cacna1h^T4306C/T4306C^* animals, suggesting that Ni^2+^ acts via additional target(s) to regulate SM formation. It will thus be interesting to examine other possible target(s) affected by Ni^2+^ using RNA sequencing or protein mass spectrometry.

A previous study shows that RhoA can regulate mRNA levels of *Cacna1h* in neonatal rat ventricular myocytes in hypoxia by mediating protein stabilization of HIF‐1α.^[^ ^47^ ^]^ However, HIF‐1α inhibition by its siRNA caused no significant changes in mRNA levels of *Cacna1h* in normoxic conditions. Thus, it appeared that RhoA mediates *Cacna1h* expression in a context dependent manner such as hypoxia. CACNA1H has been shown to facilitate Ca^2+^ influx.^[^ ^48^, ^49^ ^]^ Ca^2+^ influx has been reported to promote RhoA expression.^[^ ^38^ ^]^ Consistently, TRPV4, a calcium‐permeable cation channel, functions as a Ca^2+^ entry channel to mediate calcium influx,^[^ ^50^, ^51^ ^]^ defects in which causes reduced RhoA protein levels in ishikawa cells.^[^ ^38^ ^]^ In the present study, we also find that both loss‐of‐function mutation in *Cacna1h* (*Cacna1h^T4306C/T4306C^*) and CACNA1H inhibitor Z944 treatment lead to reduced RhoA protein levels in the tracheae. *Cacna1h* knockdown also causes reduced RhoA protein levels in human bronchial smooth muscle cells (Figure S9e,f,i, Supporting Information). It is possible that calcium channel‐mediated Ca^2+^ homeostasis is necessary for RhoA protein expression or stabilization in several contexts. However, the mechanisms underlying Ca^2+^ homeostasis‐mediated RhoA protein expression and/or stabilization remains further investigation.

Whole‐exome sequencing of five pediatric patients and their parents has revealed several mutations associated with tracheostenosis.^[^ ^3^ ^]^ However, no *SNP* in *CACNA1H* was reported to be associated with tracheostenosis in this study, possibly due to the small number of patients analyzed. It will be necessary to examine more patients with tracheostenosis including CTRD, and test for the possible association between SNP(s) in *CACNA1H* and tracheostenosis.

Finally, point mutations in *CACNA1H* are associated with several human disorders.^[^ ^52^, ^53^, ^54^ ^]^ It will also be interesting to examine whether these patients exhibit tracheal abnormalities. Our *Cacna1h* point mutant (c.4306T>C (p.Ser1436Pro)) exhibits severe congenital tracheal stenosis, providing a new model to investigate the etiology of this disease, and develop therapeutic approaches.

## 4 Experimental Section

### Experimental Animals

All animal experiments were approved by the Institutional Animal Care and Use Committees of Guangzhou Medical University and the local ethics committee (Regierungspräsidium Darmstadt, Hessen, Germany). ENU‐treated C57BL/6J male mice were obtained from Dr. Monica Justice (Baylor College of Medicine, Houston, TX). *Cacna1h* null and *Myh11‐CreER^T2^* alleles have been previously describe.^[^ ^23^, ^27^ ^]^ The *Cav3.2^flox^* allele was generated using the CRISPR‐Cas9 system and homology directed repair. Two gRNAs (targets #1 and #2) (Figure S7, Supporting Information) were selected for mouse *Cacna1h* to direct Cas9 cleavage and insertion of loxP sites flanking exon 6 using an online CRISPR design tool (http://crispr.mit.edu/). A homologous recombination vector (donor vector) constructed by using the In‐Fusion cloning method. Next, gRNAs and Cas9 mRNA were synthesized and microinjected into the cytoplasm of C57BL/6J zygotes, together with the donor vector. After injection, surviving zygotes were immediately transferred into oviducts of ICR albino pseudopregnant females. *Cacna1h^flox^* alleles were detected in G0 mice and germline‐transmitted. For genotyping of *Cacna1h* floxed mice, primers sets (loxP1‐Fwd:5′‐GAAGGGACAGACCAGTGATGAGG‐3′; LoxP1‐Rev:5′‐TCCACCTCACCACGCAGCAT‐3′) were used to generate ≈293 bp wild‐type and ≈398 bp loxP PCR amplicons. All breeding colonies were maintained under cycles of 12 h light and 12 h dark. All protocols were approved by the Institutional Animal Care and Use Committees of Guangzhou Medical University.

### Whole‐Exome Sequencing Analysis

Genomic DNA from two WT and two mutant mice were isolated using a standard protocol. Whole‐exome sequencing was conducted as previously described.^[^ ^4^ ^]^

### Alcian Blue Staining of Cartilage

Trachea cryosections were fixed in 4% paraformaldehyde for 20 min, treated with 3% acetic acid solution for 3 min, stained in 0.05% alcian blue for 10 min and counterstained with 0.1% nuclear fast red solution for 5 min. For whole‐mount staining of tracheal cartilage, dissected tracheae were fixed in 95% ethanol for 12 h followed by overnight staining with 0.03% alcian blue dissolved in 80% ethanol and 20% acetic acid. Samples were cleared in 2% KOH.

### Whole‐Mount Immunostaining

Tracheae were isolated from E12.5 to E16.5 embryos, and P0 and P60 mice. Tracheae were then fixed in 4% paraformaldehyde overnight at 4 °C and washed three times in PBS for 1 h each. Samples were incubated in 5% FBS/PBS/0.5% Triton X‐100/3% BSA for 12 h and then in primary antibodies overnight at 4 °C. After six washes in 0.5% Triton X‐100/PBS for 1 h each, samples were incubated in secondary antibodies overnight at 4 °C. Samples were washed six times in 0.5% Triton X‐100/PBS for 1 h each and mounted for imaging. To visualize smooth muscle (SM) cells and chondroblasts, tracheae were stained for αSMA and SOX9, respectively. To visualize F‐actin, tracheae were stained with 488‐conjugated phalloidin (Thermo Fisher Scientific, A12379).

### Immunostaining of Cryosections

Tracheae were dissected in PBS, fixed in 4% paraformaldehyde overnight at 4 °C, incubated in 10% sucrose and 30% sucrose for 24 h each at 4 °C, mounted in OCT embedding compound, and sectioned at 8 µm. To perform immunostaining, sections were fixed in 4% paraformaldehyde 10 min at 4 °C, followed by incubation in permeabilization solution (0.3% Triton X‐100/PBS) for 15 min at RT, incubated in blocking solution (5% FBS/PBS/3% BSA) for 1 h at RT, incubated in primary antibodies overnight at 4 °C, washed, incubated in secondary antibodies for 2 h at RT, washed, and then mounted for imaging.

### Reverse Transcription Quantitative PCR (RT–qPCR)

Total RNA extraction was conducted using a miRNeasy Mini Kit (Qiagen). cDNA was synthesized using the Maxima First Strand cDNA Synthesis Kit (Thermo Fisher Scientific), according to manufacturer's instructions. Quantitative real‐time PCR was performed using Eco Real‐Time PCR System (Illumina) and Maxima SYBR Green/Fluorescein qPCR Master Mix (Thermo Fisher Scientific). The following primers were used: *mActb* forward 5′‐CGGCCAGGTCATCACTATTGGCAAC‐3′ and *mActb* reverse 5′‐GCCACAGGATTCCATACCCAAGAAG‐3′; *mCacna1h* forward 5′‐ TCTTCATTGTCATGGCTGGCA‐3′ and *mCacna1h* reverse 5′‐ GGTCAGGTTGTTGTTCCTGACGA‐3′; *mShh* forward 5′‐GGCTGATGACTCAGAGGTGCAAAG‐3′ and *mShh* reverse 5′‐ GCTCGACCCTCATAGTGTAGAGAC‐3′; *mFgf10* forward 5′‐TTTGGTGTCTTCGTTCCCTGT‐3′ and *mFgf10* reverse 5′‐TAGCTCCGCACATGCCTTC‐3′; *mFgfr2b* forward 5′‐GCCCTACCTCAAGGTTATGAAAG‐3′ and *mFgfr2b* reverse 5′‐GATAGAATTACCCGCCAAGCA‐3′; *hCACNA1H* forward 5′‐ATGCTGGTAATCATGCTCAACTG‐3′ and *hCACNA1H* reverse 5′‐AAAAGGCGAAAATGAAGGCGT‐3′; *hGAPDH* forward 5′‐ GATTCCACCCATGGCAAATTC‐3′ and *hGAPDH* reverse 5′‐ CTGGAAGATGGTGATGGGATT‐3′.

### Western Blotting

Isolated P0 tracheae or human bronchial smooth muscle cells were lysed using RIPA buffer (Cell Signaling, 9806) supplemented with protease and phosphatase inhibitors (Cell Signaling, 5872). Lysates were centrifuged at 10 000x*g* for 10 min, subjected to SDS‐PAGE and transferred to nitrocellulose membranes. Membranes were probed with primary and HRP‐conjugated secondary antibodies (Cell Signaling Technology) and were developed using the ECL detection system (Pierce). For chemical treatment, tracheae were incubated in DMEM/F‐12 medium containing 0.1% DMSO or 2 µM Z944 for 48 h before lysis.

### Quantification of Western Blot Signals

RhoA and ACTB levels were quantified using ImageJ. RhoA levels were normalized to the values yielded by ACTB.

4.1

The following antibodies were used: mouse anti‐αSMA‐Cy3 (1:1000, Sigma–Aldrich, C6198); rabbit anti‐SOX9 (1:400, Millipore, AB5535); rat anti‐CDH1 (1:500, Santa Cruz, sc‐59778); at anti‐CDH1 (1:200, Abcam, ab11512); rabbit anti‐CACNA1H (1:400, Alomone Labs, ACC‐025); rabbit anti‐Ki67 (1:400, Cell Signaling Technologies, #9027); mouse anti‐PCNA (1:400, Santa Cruz, sc‐56); rabbit anti Cleaved Caspase‐3 (1:600, Cell Signaling Technologies, #9661); mouse anti‐acetylated α‐tubulin (1:2000, Sigma‐Aldrich, T‐7451); rabbit anti‐SCGB1A1 (1:300, Abcam; ab40873); rabbit anti‐KRT5 (1:400, Abcam; ab53121); rabbit anti‐RhoA (1:400, Cell Signaling Technologies, #2117) and rabbit anti‐ACTB (1:3000, Cell Signaling Technologies, #3700).

### Explant Culture of Mouse Embryonic Tracheae and Lungs, and Chemical Treatment

Tracheae and lungs were isolated from E16.5 embryos and cultured using an established protocol.^[^ ^55^ ^]^ For jasplakinolide (Tocris, 2792) treatment, a 300 µM stock solution was diluted to 300 nM. For Z944 (Tocris, 6367) treatment, a 2 mM stock solution was diluted to 2 µM. For NiCl_2_ (Sigma, 7718‐54‐9) treatment, a 30 mM stock solution was diluted to 30 µM. Isolated tracheae and lungs were cultured in DMEM/F‐12 medium containing the above chemicals at 37 °C in a 5% CO_2_ incubator for 48 h. For rescue experiment with Rho Activator II (Cytoskeleton, CN03), a 5 µg ml^−1^ stock solution was diluted to 5 ng ml^−1^ in DMEM/F‐12 medium and isolated tracheae and lungs were cultured for 2 h and then for another 46 h after replacing medium without Rho Activator II. For rescue experiment with jasplakinolide, isolated tracheae and lungs were cultured in DMEM/F‐12 medium containing Z944 or NiCl_2_ for 12 h and then for another 36 h after adding 300 nM jasplakinolide. 0.1% DMSO in DMEM/F‐12 medium was used as a control for Z944 and jasplakinolide treatment. 0.1% ddH_2_O in DMEM/F‐12 medium was used as a control for NiCl_2_ and Rho Activator II treatment. The medium was replaced every 24 h before collection for analysis.

### Human Bronchial Smooth Muscle Cell Culture, Chemical Treatment and siRNA Experiment

Human bronchial SM cells (LONZA, CC‐2576) were cultured in SM cell medium (ScienCell, #1101) containing 30 µM NiCl_2_ at 37 °C in a 5% CO_2_ incubator for 48 h. 0.1% ddH_2_O in the medium was used as a control. The medium was replaced every 24 h before collection for analysis. Sequences of siRNA synthesized by RiboBio (Guangzhou, People's Republic of China) for human *CACNA1H* is GCCGTGGCGTCTATGAATT.

### Ex Vivo Trachea Physiology

Spontaneous contraction of fetal tracheae was analyzed as previously described.^[^ ^26^ ^]^ Briefly, E13.5 tracheae were isolated and kept in PBS for time‐lapse imaging for 10 min every 1 second with a Zeiss LSM 800 inverted laser scanning confocal microscope. The amplitude of spontaneous contractions was defined as “1 − the ratio of tracheal smooth muscle minimum width after contraction to maximum width after relaxation”. For tracheal contractile responses elicited by acetylcholine, ≈3.5 mm sections of tracheas were isolated from P14 pups and kept in Krebs solution (127 mM NaCl, 4.7 mM KCl, 2.5 mM CaCl_2_, 1.17 mM MgSO_4_, 17 mM NaHCO_3_, 1.17 mM KH_2_PO_4_, 6.05 mM D‐glucose) aerated with carbogen at 37 °C. Tracheal rings were mounted in a wire‐myograph system (620 M; Danish Myo Technology, Aarhus, Denmark) and a resting tension of 3 mN was applied for each ring as a baseline. Contractile responses were determined by cumulative administration of indicated acetylcholine concentrations.

### RNA Sequencing and Data Analysis

Total RNA was extracted from two WT E16.5 tracheae and two *Cacna1h^T4306C/T4306C^* E16.5 tracheae, or two WT E17.5 tracheae and two *Cacna1h^T4306C/T4306C^* E17.5 tracheae using TRIzol reagent (15 596 026; Ambion, Austin, TX, USA) combined with DNase digestion to avoid contamination by genomic DNA. RNA and library preparation integrity were verified with a BioAnalyzer 2100 (Agilent) or a Qubit 2.0 (Thermofisher). 50–200 ng of total RNA was used as input for polyA enrichment followed by library preparation using Optimal Dual‐mode mRNA Library Prep Kit (BGI‐Shenzhen, China). Sequencing was performed on the MGI 2000 instrument (BGI‐Shenzhen, China), resulting in average of 20 M reads per‐library with 2 × 150 bp paired‐end setup. The raw sequencing data was filtered with SOAPnuke^[^ ^56^ ^]^ by 1) removing reads containing sequencing adapter; 2) removing reads whose low‐quality base ratio (base quality less than or equal to 15) is more than 20%; 3) removing reads whose unknown base (“N” base) ratio is more than 5%. Cleaned reads were then obtained and stored in FASTQ format. The subsequent analysis and data mining were performed on Dr. Tom Multi‐omics Data mining system (https://biosys.bgi.com). Bowtie2 was applied to align the clean reads to the gene set.^[^ ^57^ ^]^ Gene expression levels were quantified using RSEM (v1.3.1).^[^ ^58^ ^]^ Differential expression analysis was conducted using DESeq2 (v1.40.2),^[^ ^59^ ^]^ with a significance threshold set at *P* ≤ 0.05, which is appropriate for exploratory studies with a small number of differentially expressed genes. To gain insights into the biological significance of differentially expressed genes, Gene Ontology (GO) and Kyoto Encyclopedia of Genes and Genomes (KEGG) enrichment analyses were performed using the R packages clusterProfiler (v4.8.3) (DOI:10.18129/B9.bioc.clusterProfiler) and org.Mm.eg.db (v3.17.0) (Pages, H. et al. Package “AnnotationDbi”. Bioconductor Packag. Maint (2017)) in R (v4.3.1). The significance of enriched pathways was determined with a stringent *q*‐value threshold (*q* ≤ 0.05) (as shown in Table S2, Supporting Information). Pathways associated with cytoskeleton, mechanical force, and smooth muscle differentiation were specifically visualized using enrichplot (v1.20.1) (DOI:10.18129/B9.bioc.enrichplot) and ggplot2 (v3.4.3) (https://doi.org/10.1002/wics.147).

### Human Samples

Human tracheostenosis samples and healthy control tracheal tissues were provided by Guangzhou Women and Children's Medical Center Biobank (Guangzhou, China) and Cincinnati Children's Hospital Medical Center Biobank Core facility Discover together (Cincinatti, OH, USA). The clinical diagnosis of tracheostenosis was confirmed by bronchoscopic view, computed tomography scan and/or histological analysis. The study protocol and tissue usage were approved by the institutional ethics committee (number 120A01). Written informed consent was obtained from all patients’ families prior to collection of samples.

4.2

Imaging of wholemount tracheae and trachea sections was performed using a Zeiss AXIO Zoom.V16 or Zeiss 880 inverted confocal microscope, or a Leica DM6 B upright microscope. Quantification of tube width, tube length, SM area and immunofluorescence intensity was performed using ImageJ. For H&E staining, slides were baked for 30 min, deparaffinized and rehydrated, and stained with hematoxylin and eosin. Sections were visualized and photographed using a Nikon wide field microscope coupled with a DS‐Fi3 color camera.

### Statistical Analysis

Statistical analyses were performed using GraphPad software. Error bars, s.d. and *P* values were calculated by two‐tailed Student's *t*‐test. P<0.05 indicates a finding is significant. P>0.05 indicates a finding is not significant.

## Conflict of Interest

The authors declare no conflict of interest.

## Author Contributions

Z.L. and C.L. and L.M. contributed equally to this work. W.Y. conceived the project, designed experiments and analyzed data; Z.L., C.L., M.L., C.L., Y.L., X.L., H.L., Y.C., J.Z., L.L., N.B. and D.S. contributed to experiments and data analysis; W.J. and D.Y.R.S. provided infrastructure and contributed to data analysis; M.L., Z.L. and L.L. provided clinical guidance; Z.L. and W.Y. wrote the manuscript. All authors commented on the manuscript.

## Supporting information



Supporting Information

Supplemental Movie 1

Supporting Information

Supporting Information

## Data Availability

The data that support the findings of this study are available in the supplementary material of this article.
