# Supplementary material for: The T‐Type Calcium Channel CACNA1H is Required for Smooth Muscle Cytoskeletal Organization During Tracheal Tubulogenesis
Source: Adv Sci (Weinh). 2024 Oct 3;11(44):2308622. doi: 10.1002/advs.202308622 (PMC11600216; doi:10.1002/advs.202308622)
Supplement: Supplementary file 1 — Supporting Information [file ADVS-11-2308622-s003.docx]

**Supplementary Figure Legends**

**Figure S1: P0 mutant mice exhibit WT-like tracheal tube length.**

Quantification of P0 WT (n=5) and mutant (n=5) tracheal tube length. Unpaired Student’s t-test. Data are mean ± s.d.

**
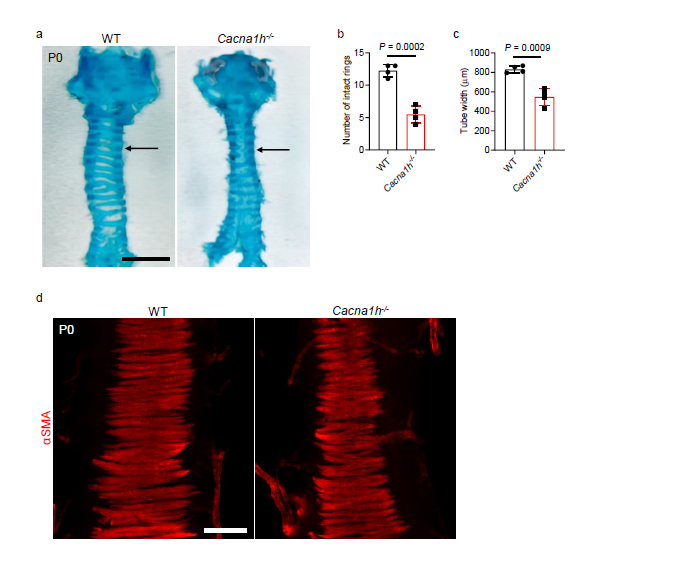
**

**Figure S2: *Cacna1h^-/-^* mice exhibit tracheal tube formation defects.**

(**a**) Representative image of ventral views of wholemount tracheae stained with alcian blue from P0 WT (n=4) and *Cacna1h^-/-^* mice (n=4). Arrows point to tracheal cartilage rings. (**b**) Quantification of the number of intact tracheal cartilage rings from P0 WT (n=4) and *Cacna1h^-/-^* (n=4) tracheae. (**c**) Quantification of P0 WT (n=4) and *Cacna1h^-/-^* (n=4) tracheal tube width. (**d**) Representative images of dorsal views of wholemount tracheae stained for αSMA (red) from P0 WT (n=4) and *Cacna1h^-/-^* mice (n=4). Scale bars: 1000 μm (**a**), 200 μm (**d**). Unpaired Student’s *t*-test. Data are mean ± s.d.

**
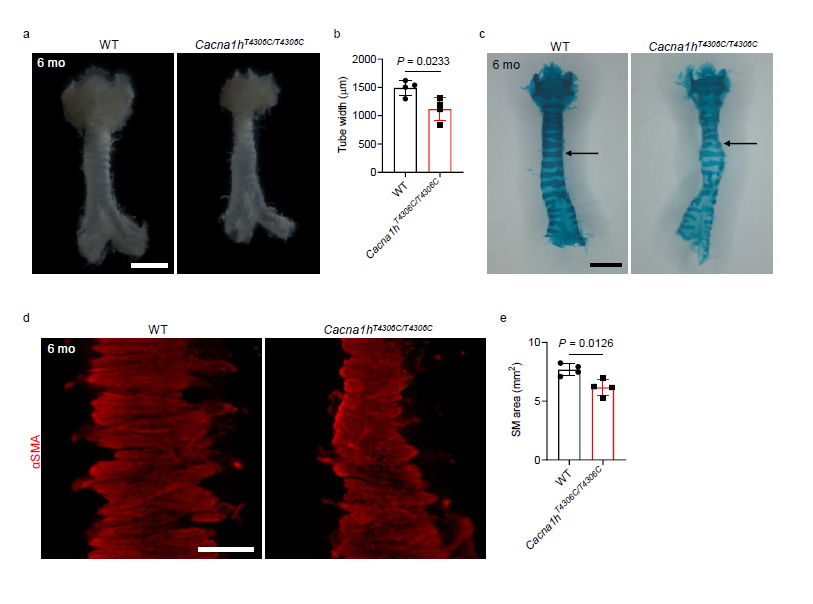
**

**Figure S3: *Cacna1h* modulates cartilage ring formation and SM organization in adult tracheas.**

(**a**) Representative images of ventral views of 6-month-old (6 mo) WT (n=4) and *Cacna1h^T4306C/T4306C^* (n=4) tracheae. (**b**) Quantification of 6 mo WT (n=4) and *Cacna1h^T4306C/T4306C^* (n=4) tracheal tube width. (**c**) Representative image of ventral views of wholemount tracheae stained with alcian blue from 6 mo WT (n=4) and mutants (n=4). Arrows point to tracheal cartilage rings. (**d**) Representative images of dorsal views of wholemount tracheae stained for αSMA (red) from 6 mo WT (n=4) and *Cacna1h^T4306C/T4306C^* mice (n=4). (**e**) Quantification of 6 mo WT (n=4) and *Cacna1h^T4306C/T4306C^* (n=4) tracheal SM area. Scale bars: 2 mm (**a, c**), 500 μm (**d**). Unpaired Student’s *t*-test. Data are mean ± s.d. mo, month.

**
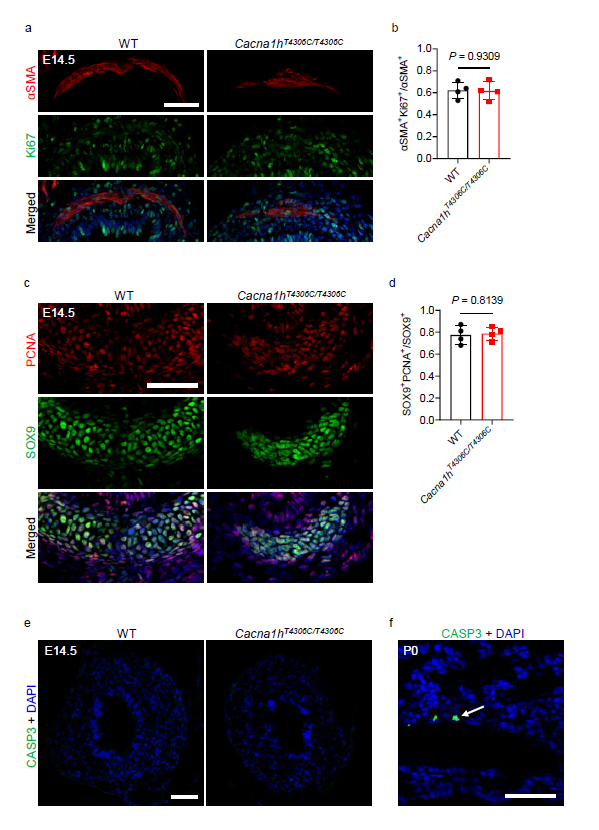
**

**Figure S4: *Cacna1h^T4306C/T4306C^* tracheae display WT-like proliferation and apoptosis rate of SM cells or SOX9^+^ mesenchymal cells.**

(**a**) Immunostaining for αSMA (red) and Ki67 (green) and DAPI staining (blue) of transverse sections of E14.5 WT (n=4) and *Cacna1h^T4306C/T4306C^* (n=4) tracheae. (**b**) Percentage of αSMA^+^ cells that are Ki67^+^. (**c**) Immunostaining for PCNA (red) and SOX9 (green) and DAPI staining (blue) of transverse sections of E14.5 WT (n=4) and *Cacna1h^T4306C/T4306C^* (n=4) tracheas. (**d**) Percentage of SOX9^+^ cells that are PCNA^+^. (**e**) Immunostaining for cleaved caspase-3 (CASP3, green) and DAPI staining (blue) of transverse sections of E14.5 WT (n=4) and *Cacna1h^T4306C/T4306C^* (n=4) tracheae. (**f**) Immunostaining for CASP3 (green) and DAPI staining (blue) of P0 WT lung tissue sections (n=5) as a positive control. Arrows point to apoptotic cells. Scale bars: 50 μm. Unpaired Student’s *t*-test. Data are mean ± s.d.

**
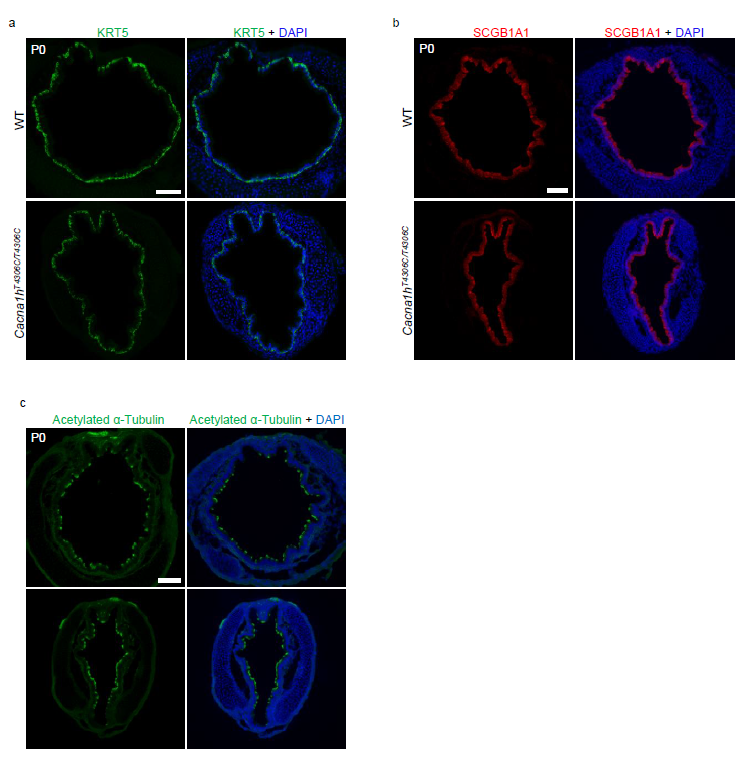
**

**Figure S5: *Cacna1h^T4306C/T4306C^* tracheae display WT-like differentiation of basal cells, club cells and multiciliated cells.**

(**a**) Immunostaining for KRT5 (green) and DAPI staining (blue) of transverse sections of P0 WT (n=3) and *Cacna1h^T4306C/T4306C^* (n=3) tracheae. (**b**) Immunostaining for SCGB1A1 (red) and DAPI staining (blue) of transverse sections of P0 WT (n=3) and *Cacna1h^T4306C/T4306C^* (n=3) tracheae. (**c**) Immunostaining for acetylated α-Tubulin (green) and DAPI staining (blue) of transverse sections of P0 WT (n=3) and *Cacna1h^T4306C/T4306C^* (n=3) tracheae. Scale bars: 50 μm.

**
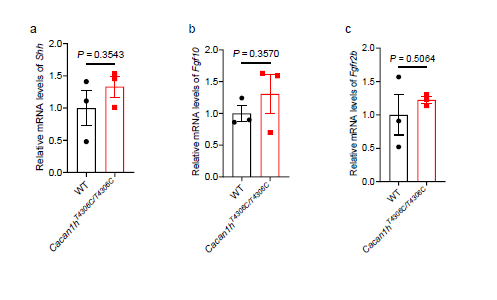
**

**Figure S6: *Cacna1h^T4306C/T4306C^* tracheae exhibt no significant changes in expression levels of *Shh*, *Fgf10* or *Fgfr2b*.**

(**a**) qPCR analysis of *Shh* mRNA levels in E16.5 WT (n=3) and *Cacna1h^T4306C/T4306C^* (n=3) tracheae. (**b**) qPCR analysis of *Fgf10* mRNA levels in E16.5 WT (n=3) and *Cacna1h^T4306C/T4306C^* (n=3) tracheae. (**c**) qPCR analysis of *Fgfr2b* mRNA levels in E16.5 WT (n=3) and *Cacna1h^T4306C/T4306C^* (n=3) tracheae.

**
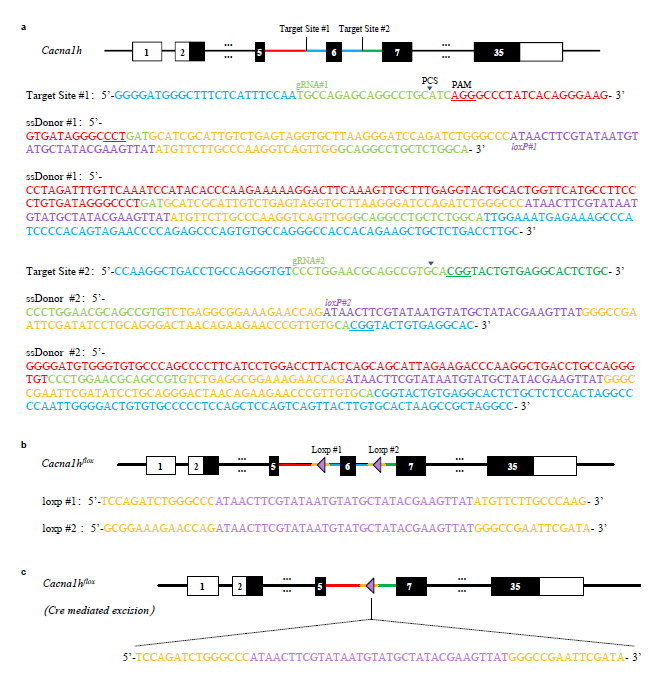
**

**Figure S7: Generation of *Cacna1h^flox^* mice.**

(**a**) The CRISPR/Cas9 system was used to generate mice carrying *loxP* sites flanking exon 6 of *Cacna1h*. gRNA #1 and gRNA #2 were used to target 303 bp and 301 bp single stranded donor sequences (ssDonor #1 and #2) carrying *loxP* sites to intronic sites flanking exon 6, respectively. (**b**) One mouse carrying two *loxP* sites in *cis* (*Cacna1h^flox^*) was recovered, and sequences across the two *loxP* sites following germline transmission of the allele are shown. (**c**) Cre expression in mice carrying the floxed *Cacna1h* allele results in a 1.3 kb deletion with a single residual *loxP* site remaining.

**
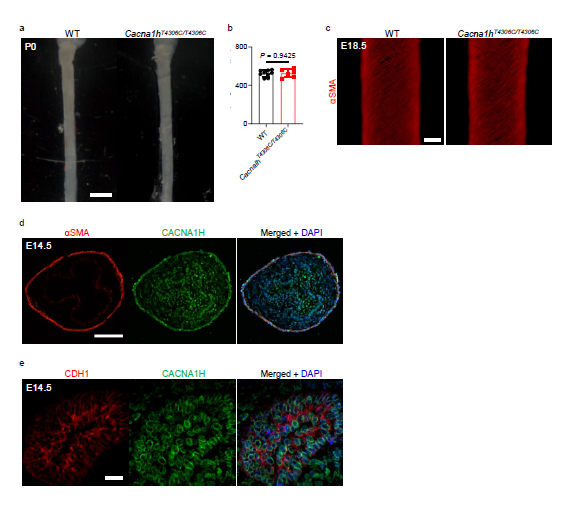
**

**Figure S8: *Cacna1h^T4306C/T4306C^* mice display no obvious defects in esophageal expansion** **or esophageal SM organization.**

(**a**) Representative images of P0 WT (n=9) and *Cacna1h^T4306C/T4306C^* (n=6) esophagi. (**b**) Quantification of P0 WT (n=9) and *Cacna1h^T4306C/T4306C^* (n=6) esophageal width. (**c**) Immunostaining for αSMA (red) in E18.5 WT (n=9) and *Cacna1h^T4306C/T4306C^* (n=6) esophagi. (**d**) Immunostaining for αSMA (red) and CACNA1H (green) and DAPI staining (blue) of transverse sections of E14.5 WT esophagi (n=5). (**e**) Immunostaining for CDH1 (red) and CACNA1H (green) and DAPI staining (blue) of transverse sections of E14.5 WT esophagi (n=5). Scale bars: 1000 µm (**a**), 100 μm (**c, d**), 20 μm (**e**). Unpaired Student’s *t*-test. Data are mean ± s.d.

**
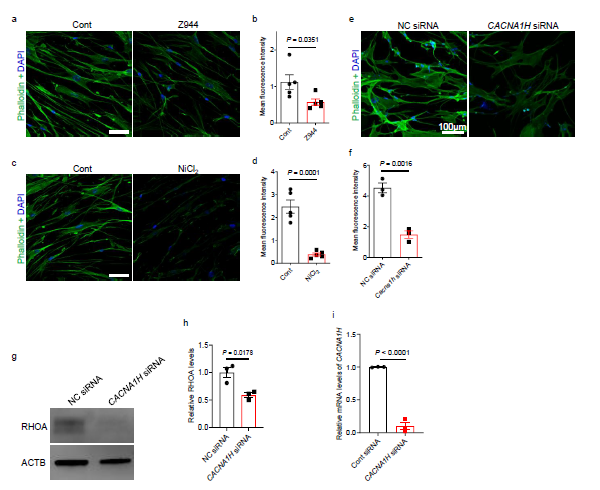
**

**Figure S9: Z944 or** **NiCl_2_ treatment leads to reduced F-actin levels in human bronchial SM cells.**

(**a**) Phalloidin (green) and DAPI (blue) staining in human bronchial SM cells after a 48 hour DMSO or 2 μM Z944 treatment. (**b**) Quantification of mean phalloidin fluorescence intensity in human bronchial SM cells after a 48 hour DMSO or 2 μM Z944 treatment. (**c**) Phalloidin (green) and DAPI (blue) staining in human bronchial SM cells after a 48 hour ddH_2_O or 30 μM NiCl_2_ treatment. (**d**) Quantification of mean phalloidin fluorescence intensity in human bronchial SM cells after a 48 hour ddH_2_O or 30 μM NiCl_2_ treatment. (**e**) Phalloidin (green) and DAPI (blue) staining in 48-hour NC siRNA or *CACNA1H* siRNA-transfected human bronchial SM cells. (**f**) Quantification of mean phalloidin fluorescence intensity in 48-hour NC siRNA or *CACNA1H* siRNA-transfected human bronchial SM cells. (**g**) Western blotting for RHOA and ACTB in 48-hour NC siRNA or *CACNA1H* siRNA-transfected human bronchial SM cells. (**h**) Quantification of relative RHOA levels in 48-hour NC siRNA or *CACNA1H* siRNA-transfected human bronchial SM cells. NC, Negative control. (**i**) Quantification of relative mRNA levels of *CACNA1H* in 48-hour NC siRNA or *CACNA1H* siRNA-transfected human bronchial SM cells. NC, Negative control. Scale bars: 50 µm (**a, c**), 100 µm (**e**). Unpaired Student’s *t*-test. Data are mean ± s.d.

**
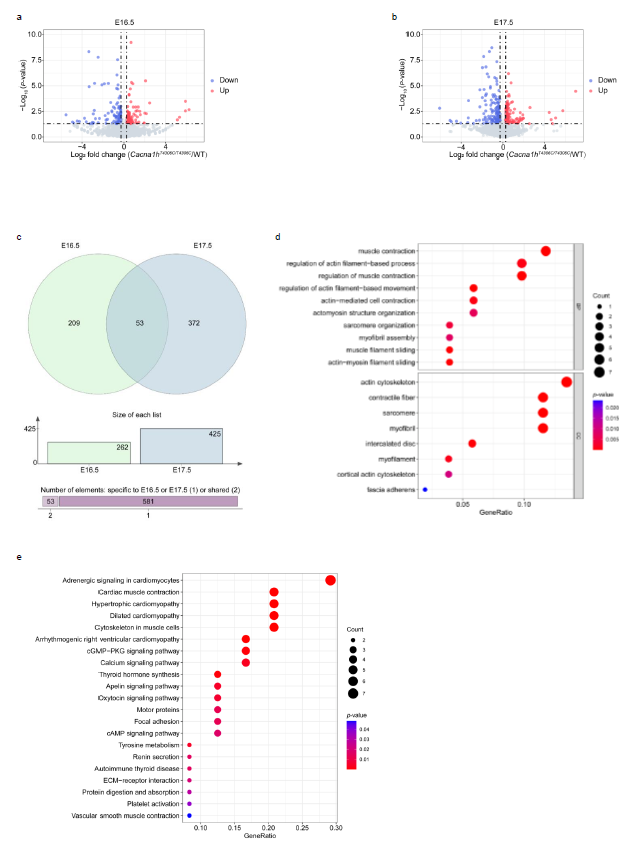
**

**Figure S10: RNA-Seq analysis of gene expression changes in E16.5 and E17.5 *Cacna1h^T4306C/T4306C^* tracheae.**

(**a**) Differentially expressed genes in E16.5 *Cacna1h^T4306C/T4306C^* tracheae compared with WT by RNA-Seq. (**b**) Differentially expressed genes in E17.5 *Cacna1h^T4306C/T4306C^* tracheae compared with WT by RNA-Seq. (**c**) Analysis of the number of differentially expressed genes shared in E16.5 and E17.5 *Cacna1h^T4306C/T4306C^* tracheae. (**d**) Gene Ontology classification of differentially expressed genes shared in E16.5 and E17.5 *Cacna1h^T4306C/T4306C^* tracheae. (**e**) Kyoto Encyclopedia of Genes and Genomes classification of differentially expressed genes shared in E16.5 and E17.5 *Cacna1h^T4306C/T4306C^* tracheae. RNA-Seq, RNA sequencing; CC, Cellular component; BP, Biological process.

**Supplemental table 3: Healthy control and tracheostenosis patient characteristics.**

| Samples | Age (day, month or year) | Gender | Other conditions | CTRD-related Surgical Therapy |
| --- | --- | --- | --- | --- |
| Control 1 | 2 years | Female | None | None |
| Control 2 | 2 years | Female | None | None |
| Control 3 | 5 months | Male | None | None |
| Control 4 | 4 years | Male | None | None |
| Control 5 | 4 months | Male | None | None |
| Control 6 | 5 months | Female | None | None |
| Tracheostenosis | 2 years | Female | PAS | STP, PAS repair |
| Tracheostenosis | 1 year | Male | PAS | STP, PAS repair |
| Tracheostenosis | 1 year | Male | PAS | STP, PAS repair |
| Tracheostenosis | 4 years | Female | None | STP |
| Tracheostenosis | 8 months | Male | PAS | STP, PAS repair |
| Tracheostenosis | 2 months | Male | PAS | STP, PAS repair |
| Tracheostenosis | 5 months | Female | None | STP |

PAS = pulmonary artery sling. STP = slide tracheoplasty.

**Supplementary Movie**

Compared to WT tracheae (n=5), *Cacna1h^T4306C/T4306C^* tracheae (n=5) exhibit reduced amplitude of spontaneous contractions at E13.5.
